# Transcriptional profiling of *Salmonella enterica* serovar Enteritidis exposed to ethanolic extract of organic cranberry pomace

**DOI:** 10.1371/journal.pone.0219163

**Published:** 2019-07-03

**Authors:** Quail Das, Dion Lepp, Xianhua Yin, Kelly Ross, Jason L. McCallum, Keith Warriner, Massimo F. Marcone, Moussa S. Diarra

**Affiliations:** 1 Department of Food Science, University of Guelph, Ontario, Canada; 2 Guelph Research and Development Centre, Agriculture and Agri-Food Canada, Guelph, Ontario, Canada; 3 Summerland Research and Development Center, Agriculture and Agri-Food Canada, Summerland, British Columbia, Canada; 4 Charlottetown Research and Development Center, Agriculture and Agri-Food Canada, Charlottetown, Prince Edward Island, Canada; University of Minnesota, UNITED STATES

## Abstract

Non-typhoidal *Salmonella enterica* serovars continue to be an important food safety issue worldwide. Cranberry (*Vaccinium macrocarpon Ait*) fruits possess antimicrobial properties due to their various acids and phenolic compounds; however, the underlying mechanism of actions is poorly understood. We evaluated the effects of cranberry extracts on the growth rate of *Salmonella enterica* serovars Typhimurium, Enteritidis and Heidelberg and on the transcriptomic profile of *Salmonella* Enteritidis to gain insight into phenotypic and transcriptional changes induced by cranberry extracts on this pathogen. An ethanolic extract from cranberry pomaces (KCOH) and two of its sub-fractions, anthocyanins (CRFa20) and non-anthocyanin polyphenols (CRFp85), were used. The minimum inhibitory (MICs) and bactericidal (MBCs) concentrations of these fractions against tested pathogens were obtained using the broth micro-dilution method according to the Clinical Laboratory Standard Institute’s guidelines. Transcriptional profiles of *S*. Enteritidis grown in cation-adjusted Mueller-Hinton broth supplemented with or without 2 or 4 mg/ml of KCOH were compared by RNASeq to reveal gene modulations serving as markers for biological activity. The MIC and MBC values of KCOH were 8 and 16 mg/mL, respectively, against all tested *S*. *enterica* isolates. The MIC value was 4 mg/mL for both CRFa20 and CRFp85 sub-fractions, and a reduced MBC value was obtained for CRFp85 (4 mg/ml). Treatment of *S*. Enteritidis with KCOH revealed a concentration-dependent transcriptional signature. Compared to the control, 2 mg/ml of KCOH exposure resulted in 89 differentially expressed genes (DEGs), of which 53 and 36 were downregulated and upregulated, respectively. The upregulated genes included those involved in citrate metabolism, enterobactin synthesis and transport, and virulence. Exposure to 4 mg/ml KCOH led to the modulated expression of 376 genes, of which 233 were downregulated and 143 upregulated, which is 4.2 times more DEGs than from exposure to 2 mg/ml KCOH. The downregulated genes were related to flagellar motility, *Salmonella* Pathogenicity Island-1 (SPI-1), cell wall/membrane biogenesis, and transcription. Moreover, genes involved in energy production and conversion, carbohydrate transport and metabolism, and coenzyme transport and metabolism were upregulated during exposure to 4 mg/ml KCOH. Overall, 57 genes were differentially expressed (48 downregulated and 9 upregulated) in response to both concentrations. Both concentrations of KCOH downregulated expression of *hilA*, which is a major SPI-1 transcriptional regulator. This study provides information on the response of *Salmonella* exposed to cranberry extracts, which could be used in the control of this important foodborne pathogen.

## Introduction

Despite control measures and efforts deployed in food production continuum, non-typhoidal *Salmonella enterica* serovars continue to rank among the most common causes of bacterial gastroenteritis worldwide [1, 2]. According to the USA Foodborne Diseases Active Surveillance Network (FoodNet), *Salmonella* is one of the major zoonotic pathogens, causing 2,074 hospitalizations and 32 death in the USA in 2015 [3, 4]. In Canada, *S*. Enteritidis, *S*. Typhimurium and *S*. Heidelberg were among the top *Salmonella* serovars involved in human salmonellosis in 2017 [5]. In the last 10 years, the incidence of *Salmonella* Enteritidis (SE) has increased three-fold in Canada, and a similar increase of this serovar isolate from poultry sources has been documented nationally during the same timeframe [2]. Most frequently, *Salmonella enterica* serovars asymptomatically colonize the alimentary tracts of poultry. However, some serovars have been isolated in young chicks with systemic infections that may further lead to the infection of egg contents in mature birds by migration through the egg shells and membranes [6]. Among the non-typhoidal *Salmonella*, SE is the most invasive associated with poultry reproductive tissues. In Canada, the overall proportion of SE identified among retail chicken breast samples increased significantly from 8% in 2015 to 17% in 2016 [5].

The mechanisms by which non-typhoidal *Salmonella* survives in, colonizes and invades the environment and their hosts have been the subject of various studies [7]. To adapt to hostile environments, *Salmonella* must constantly deploy strategies to counteract various stress conditions, such as changing pH (from 3.99 to 9.5) and temperatures (high as 54°C or low as 2°C), high osmotic pressure, low oxygen availability, and the presence of salts (concentrations up to 4% w/v) and other antimicrobial compounds (peptides) that constantly challenge the adaptability of this pathogen [8]. Several factors including outer membrane components (proteins and lipopolysaccharides) as well as fimbriae and flagella play important roles in both colonization and systemic infection by *Salmonella* inside a broad range of animal and human hosts. The persistence of *Salmonella* may be in part due to the presence of the invasin gene (*inv*), which enables this bacterium to colonize host intestinal tissues [9]. *Salmonella* carries a large number (> 92) of fimbrial and non-fimbrial genes associated with adhesion. Previously, our research group found the fimbrial operons *fimACDHFI* (type 1 fimbriae), *lpfABCDE* (long polar fimbriae), *agf/csgABCEFG* (thin aggregative fimbriae), *stfACDEFG* and *sthABCDE* in serovars Enteritidis, Typhimurium, Heidelberg, Hadar and Kentucky isolated from chicken [10]. It has been reported that *S*. Typhimurium mutants lacking genes encoding the potassium binding and transport protein *kdpA*, hypothetical protein *yciG*, flagellar hook cap protein *flgD*, and nitrate reductase subunit *narZ* were significantly deficient in their acid tolerance responses and displayed variations in their virulence characteristics [11]. The type III secretion systems (T3SSs), T3SS1 and T3SS2, are encoded by the pathogenicity islands (SPI-1 and SPI-2, respectively), which play a central role in *Salmonella*-host interactions [12]. While SPI-I is required for invasion into host epithelial cells, SPI-II is required for intracellular survival and the systemic phase of infection [13].

Iron is an essential element for growth and survival for almost all aerobic microorganisms, including *Salmonella*. Whole genomic analysis revealed that *Salmonella* are well equipped with iron uptake genetic determinants such as genes encoding the FeoA and FeoB proteins (involved in uptake of soluble Fe^2+^); the catecholate siderophore enterobactin synthesis and utilization genes including the Fe^3+^-siderophore outer membrane receptors *cirA*, f*epA*, and transporters *fepBCDEG;* the hydroxamate siderophore transport genes *fhuABCD* and ferric-siderophore transport periplasmic protein complex TonB, ExbB and ExbD [10]. We recently observed the importance of catecholate-iron and hydroxamate-iron uptake system of both *S*. Enteritidis and *S*. Typhimurium when grown in iron-restricted media [14].

Multi-antimicrobial resistant *Salmonella* strains isolated from both humans and livestock [2, 7, 15] have the potential to spread to and persist in the natural environment, farm workers, and food processing facilities. Identifying solutions to mitigate antimicrobial resistance while controlling pathogenic bacteria such as *Salmonella* will require researchers to address several scientific needs, including decreasing survival and growth of pathogens to lessen the risks posed by them.

Several alternative strategies, including the use of phytochemicals, to control both gram-negative and gram-positive pathogens have been investigated [16–18]. Fruits of American cranberry (*Vaccinium macrocarpon Ait*.) are a source of bioactive polyphenolic compounds having a wide range of biological activities, including antioxidant, antimicrobial, and anti-inflammatory [15, 19]. Cranberry extracts have been reported to affect bacterial cell surface structure and integrity, damage inner membranes, and affect the iron uptake system [18, 20]. Ethanol extract organic cranberry pomace showed 3–4 times the phenolic acids, tartaric esters, and antioxidant activities of the juice itself, while flavonols and anthocyanins were increased by 5 and 6 times, respectively [19, 21]. At 1 mg/ml, cranberry pomace extracts against *Staphylococcus aureus* [including methicillin resistance strain (MRSA)], induced a transcriptional signature similar to that of peptidoglycan-acting antibiotics by upregulating *vraR/S*, *murZ*, *lytM*, *pbp2*, *sgtB*, *fmt* genes [22].

In this study, we aimed to investigate the antibacterial activities of an ethanolic extract from cranberry pomace (KCOH) and two of its sub-fractions (anthocyanins: CRFa20 and non-anthocyanin polyphenols: CRFp85) against different serovars of *Salmonella* and to explore the signature of KCOH on the transcriptional profile in *S*. Enteritidis to unveil overall patterns of gene expression.

## Materials and methods

### Cranberry extracts

The ethanolic extract of cranberry pomace (KCOH) was prepared from organic cranberry (*V*. *macrocarpon*) pomace (CP) as previously described [19]. Briefly, phenolic-rich compounds were extracted with 80% ethanol from the CP. After extraction, ethanol was removed by rotary evaporation and the remaining extracts were freeze-dried at -30°C for 10–11 days to generate crude pomace extract (KCOH) and stored at -20°C until testing.

### Sub-fractions

A total of 1 g of KCOH dried powder was dissolved in Milli-Q water and fractionated by flash chromatography (Buchi Sepacore X-50, Buchi, Geneva, Switzerland) using 100 g Redi-Sep RF high-performance Gold C18 columns (230 mm x 15 mm; 20–40 um particle size; 87.7 ml column volume) (Teledyne Isco, Lincoln, NE, USA) [23]. Briefly, the crude mixture was loaded onto a preconditioned column (95% water; 5% methanol), and 10 column volumes (CVs) of water were initially applied to elute sugars and small organic acids, including citric acid. Next, 10-CVs of ethyl acetate was used to elute non-anthocyanin polyphenolics to generate the phenolic fractions (CRFp) [24]. Finally, 5-CVs of acidified methanol (0.01% HCl) was used to elute anthocyanins (CRFa). Both sub-fractions Fp and Fa were dried down by a combination of rotary evaporation (Heidoloph Instruments, Schwabach, Germany) and freeze drying (LabConco Corporation, Kansas City, MO, USA). Fp was subsequently resuspended as a slurry in MilliQ water, and further fractionated using the flash chromatography system described above. In brief, CRFp was sequentially washed with 10 CVs of 15% methanol, 85% methanol, and 100% ethyl acetate, generating subfractions CRFp15, CRFp85 and CRFp100. Similarly, CRFa was resuspended in MilliQ water and further fractionated using the flash chromatography system described above. In brief, CRFa was sequentially washed with 10 CVs of 20% methanol, 85% methanol, and 100% methanol, generating sub-fractions CRFa20, CRFa85 and CRFa100. All resultant sub-fractions were dried down using a combination of rotary evaporation and freeze drying. From 1 gram of starting material, 90mg of CRFa20 and 425mg of CRFp85 were obtained.

The quality of sub-fractions was evaluated by 1H NMR methods (Bruker Avance III 600 NMR spectrometer, Bruker Biospin Ltd., Milton, ON, Canada), prior to antimicrobial screening, to account for possible crystallized water, organic solvents, or hydrolyzed C18 stationary phases. Based on antimicrobial screening results, 2 sub-fractions of interest (CRFa20, CRFp85) were selected for further characterization by UPLC-MS/MS [23]. Commercial standards were used to create seven-point calibration curves of integrated area under the curve versus concentration, and peaks were reported as standard equivalents. Anthocyanins were quantified using cyanidin-3-O-glucoside (Polyphenols AS, Sandnes, Norway). Flavonols, iridoids, procyanidins and condensed tannins contents were determined using Quercetin-3-O-galactoside (Sigma-Aldrich Canada Co, Oakville, ON); monotropein (Sigma-) and Procyanidin B2 (Extrasynthese, Genay Cedex, France), respectively. Hydroxycinnamic content was quantified using 3-*O*-caffeoylquinic acid (chlorogenic acid) (Chromadex, Irvine, CA, USA). For each extract, the dry weight for each peak was calculated. Reported values represent the averages of the duplicate samples. Due to complexity of the sample matrix and a lack of available standards, most peaks were only tentatively identified using a combination of UV-vis absorption characteristics, chromatographic retention behaviour, MS scans and follow up MS/MS experiments, along with comparisons to previously reported HPLC-MS studies of cranberries.

KCOH and its two sub-fractions (CRFp85 and CRFa20) were dissolved in 80% methanol at a stock concentration of 128 mg/ml, filter sterilized through a sterile cellulose acetate filter assembly (pore size, 0.2 mm) and stored at -20°C until used in antimicrobial assays.

### Bacterial strains and culture conditions

Six different strains of *S*. *enterica* serovars: Typhimurium (SALH-394-2-1893 and monophasic ABBSB1218-1-3128), Enteritidis (ABBSB1004-1-3180 and ABB07-SB3071-3346), and Heidelberg (SALB-159-4-1773 and ABB07-SB3031-3342) isolated from broiler were used in this study [10]. All Typhimurium and Heidelberg isolates were multi-drug resistant to amoxicillin-clavulanic acid, ceftiofur, ceftriaxone, ampicillin and cefoxitin [10, 25]. *Escherichia coli* ATCC 25922 was used as quality control. Bacteria from frozen stocks at -80°C were grown on Mueller Hinton Agar (MHA) or Cation-Adjusted Mueller-Hinton Broth (CAMHB: Becton Dickinson, Mississauga, ON). Colony forming units per ml (CFU/ml) were determined by viable bacterial counts on Tryptic Soy Agar (TSA, Becton Dickinson). For growth studies, a single colony of each strain was inoculated into 5 ml CAMHB and the cultures were incubated at 37°C with agitation (200 rpm) for 20 h.

### Determination of minimum inhibitory concentrations (MICs)

The MICs were determined by a broth micro-dilution method in CAMHB using *E*. *coli* ATCC 25922 as the quality control according to the Clinical Laboratory Standard Institute’s (CLSI’s) guidelines. The stock solution (128 mg/ml) of sterile KCOH, CRFa20 and CRFp85 were diluted by two-fold serial dilutions using CAMHB in a honeycomb 100-well microtiter plates (Oy Growth Curves Ab Ltd, Helsinki, Finland) in a concentration ranging from 0 to 32 mg/ml; followed by the addition of 100 μl of overnight cultures of *Salmonella* strains in each well at a final concentration of 10^5^ CFU/ml, resulting in a final well volume of 200 μl. Plates were loaded in a BioscreenC (Growth Curves USA, Piscataway, NJ) for 24 h incubation at 37°C to automatically record the optical densities at 600 nm (OD_600nm_) every 20 min. Media without inoculum, but with the studied concentrations of tested products, were included as blanks. The OD with blanks were subtracted from the OD of inoculated wells containing equivalent concentrations of studied products. The OD data were used to produce OD/time plots using Microsoft Excel. The MIC was determined as the minimum concentration of KCOH, CRFa20 or CRFp85 at which no increase in optical density was observed over 24 h. Ceftiofur (Sigma Aldrich) was used as a control antibiotic. Minimal Bactericidal Concentration (MBC) was determined after 24 h-growth, as the concentration of tested products needed to kill at least 99.9% of the initial inoculums as determined by plating 100 μl of each well with no visible growth on the MHA after an overnight incubation at 37°C. Viable cell populations were estimated using the spread plate method after 24 h incubation.

### RNA extraction and purification

An overnight culture of *S*. Enteritidis ABBSB1004-1-(3180) was diluted with fresh CAMHB and grown in an orbital shaker (225 rpm) at 37°C until the logarithmic phase (OD_600nm_ of 0.6 = approximately 1 x 10^8^ cfu/ml) was reached. The culture was then divided into three flasks and KCOH was added to a final concentration of 0, 2, and 4 mg/ml. Bacterial cells in all three conditions were incubated with agitation at 37°C for 4-hours followed by the centrifugation at 6,000 X g for 10 min at room temperature. The pellets were then kept with RNA*later* Stabilization Solution (ThermoFisher Scientific) for 24 h at 4°C, washed with ice-cold 1xPBS and re-centrifuged again at 6,000 X g for 10 min. Total RNA was extracted using the PureLink RNA Mini Kit (Ambion, Invitrogen Fisher Scientific, Carlsbad, CA, USA) according to the manufacturer’s instructions. The RNA concentration and purity were determined using a Nanodrop 2000c (Thermo Scientific) and the integrity of the extracted RNA was examined using Agilent Bioanalyzer RNA Nano assay (Agilent Technologies, CA, USA). Sample with RNA integrity number (RIN) > = 8 was used for library construction. RNA (2 μg) was treated with 2U Turbo DNase (Invitrogen) at 37°C for 30 min and four biological replicates from each treatment group was used for RNA-Seq analysis.

### RNA-Seq library preparation

Bacterial stranded mRNA sequencing libraries were constructed with the TruSeq Stranded mRNA Library Prep Kit (Illumina, RS-122-9004DOC) according to the manufacturer’s instruction. Briefly, mRNA was enriched from total RNA by rRNA depletion using the Ribo-Zero rRNA removal kit (Illumina) and then fragmented and primed for cDNA synthesis. First-strand cDNA was synthesized with 200 U Superscript II reverse transcriptase (Invitrogen) at 25°C for 10 min, 42°C for 15 min, 70°C for 15 min, followed by second-strand synthesis with DNA Polymerase I and RNase H, incorporating dUTP in the Second Strand Marking Mix (SMM), at 16C for 1h. Following clean-up of double-stranded cDNA with AMPure beads (Beckman Coulter), the 3’ ends were adenylated and adapter ligation was performed to add barcodes and p5 and p7 primers to each end. Ligation reactions were purified by two rounds of AMPure bead clean-up, and the ligated fragments were enriched by PCR for 15 cycles at 98°C for 10s, 60°C for 30s and 72°C for 30s, followed by clean-up with AMPure beads. Size distributions of purified libraries were assessed with a Bioanalyzer 2100 (Agilent) using the DNA High Sensitivity kit (Agilent) according to the manufacturer’s instructions and quantified with the Qubit dsDNA HS Kit (Fisher). Libraries were pooled at 10 nM equimolar concentrations and sequenced on a MiSeq instrument (Illumina) using a 150-cycle v3 reagent kit (Illumina).

### RNA-Seq data analysis

Raw reads from the sequenced libraries were quality-filtered and differential gene expression analysis was performed following the SPARTA workflow [26]. Briefly, read-trimming and adaptor-removal was performed with Trimmomatic [27]. The filtered reads were mapped to the *Salmonella* Enteritidis strain ABBSB1004-1 genome (GCA_000973935.1) using bowtie v1.1.1 and the counts per feature were determined with the htseq-count tool within HTSeq v0.6.1 [28]. Differential gene expression analysis was performed in R v3.2.5 with the edgeR package v3.6.8 [29]. Genes with an absolute log_2_-fold change of   > 2 and false discovery rate (FDR) *<* 0.05 in (A) control vs cranberry 2 mg/mL; (B) control vs cranberry 4 mg/mL were considered differentially expressed. Clusters of orthologous groups (COGs) were assigned to the *Salmonella* Enteritidis ABBSB1004-1 predicted proteins using NCBI COG software (April 2012 release) [30].

### Validation by reverse transcription (RT)-quantitative PCR (RT-qPCR) assay

Seven genes that showed significant up- or down-regulation were selected for quantitative reverse transcription-PCR (RT-qPCR) validation. Total RNA was isolated as described above and reverse transcribed to cDNA using High Capacity RNA-to-cDNA Kit (Applied Biosystems 4387406). All primers were designed with Primer 3 software with an amplicon size of approximately 100 to 138 bp. The efficiency of the primers was checked by melting curve and standard curve analysis. Gene names and primer sequences used for RT-qPCR are presented in Table 1. The reaction mixture contained 20 μL final volume with 1 μL of cDNA (1:10 dilution), 3 μL of each primer (1 mM), 3 μL of nuclease-free water and 10 μL of Universal SYBR Green Supermix (BioRad, Canada). The RT-qPCR assay was performed on the 7500 Real-time PCR System (Applied Biosystems, Foster, USA). The amplification program was as follows: one cycle at 95°C for 3:20 min, and 40 cycles at 95°C for 30 s, 58°C for 30 s, and 72°C for 30 s. The 2^-ΔΔCt^ method was used to analyze the relative gene expression data, using *rpoD* as the reference gene for normalization. All RT-qPCR assays were conducted using three biological and three technical replicates.

**Table 1:** List of primers used in this study.

Gene	Primer sequence (5'-3'): Forward	Primer sequence (5'-3'): Reverse	Product length (bp)
***hilA***	ATCGTCGGGAGTTTGCTATT	ACTGACCAGCCATGAAAAGA	100
***fliA***	AGTATCGTCAGATGTTGCTC	GATGTTCTTCAGTCACCAGT	105
***NnarH***	TTACGACTACCAGAACCTGC	GCCCGCTAGTAATTTTGTCC	110
***invH***	AGAGCAACTCATGACCGAAT	TCTTTCATGGGCAGCAAGTA	129
***entH***	TGGAAACGGCATTTAACGCT	CCGCCATTGATCCTAACGTC	122
***rpoD***	ACATCGCTAAACGTATCGAA	GTACTGTTCCAGCAGATAGG	135
***car***	ATCTCTTTTTGGCATAGGGG	ACGTCGTTATTAATGCGGTA	126
***ssaT***	AGCAGGTAATGATGATGGCA	ATAACTTCTACCTGGTGGCG	138

### Statistical analysis

All experiments were performed in three separate assays, each including two technical replicates. The RNA-seq differential expression between control group and treatment with 2 mg/ml and 4 mg/ml KCOH were analyzed with the Bioconductor package edgeR which is analogous to t- and F-statistics tools [29] using a generalized linear model (glm) and the likelihood ratio test. A log_2_-fold change of   > 2 and false discovery rate (FDR) cutoffs ≤ 0.05 were used. The false discovery rates were calculated using the Beniamini-Hochberg method [31].

## Results

### Composition of two fractions of cranberry

**Fig 1:**
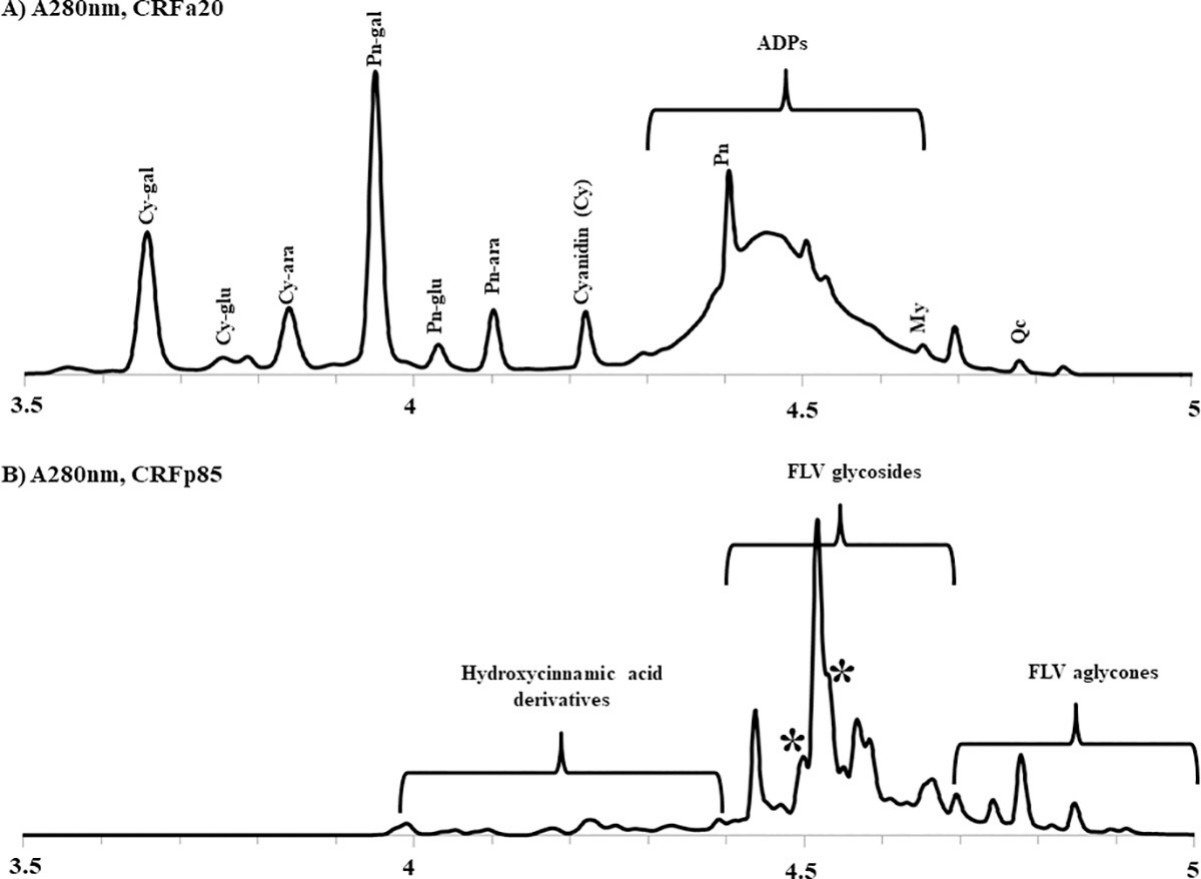
**(a)** Anthocyanin-enriched (CRFa20) and **(b)** Flavonol-enriched (CRFp85) sub-fractions of KCOH. Cy = cyanidin; Pn = Peonidin; ADP = anthocyanin derived pigments; My = myricetin; Qc = quercetin; gal = 3-O-galactopyranoside; glu = 3-O-glucopyranoside; ara = 3-O-arabinopyranoside; FLV = flavonols; * = coumaroylated iridoids.

The two major sub-fractions of interest enriched for anthocyanins (CRFa20) and flavonols (CRFp85) prepared from KCOH were analyzed by UPLC-MS. CRFa20 was composed of 27.6% anthocyanins, 43% anthocyanin derived pigments and 0.7% flavonol aglycones by mass. The tentative structures of the anthocyanin derived pigments are consistent with oligomers built from condensation reactions between proanthocyanindins with anthocyanin glycosides, principally peonidin-3-O-galactoside and cyanidin-3-O-galactoside. On the other hand, CRFp85 was mainly composed of 37.8% flavonols, primarily quercetin-3-O-galactoside along with 0.2% anthocyanins; 2.1% hydroxycinaminic acids; 9.6% acylated monoproteins; and 15.6% procyanidins (catechin) derivatives (Fig 1).

### Effect of cranberry pomace extracts on the growth of *S*. *enterica*

**Fig 2:**
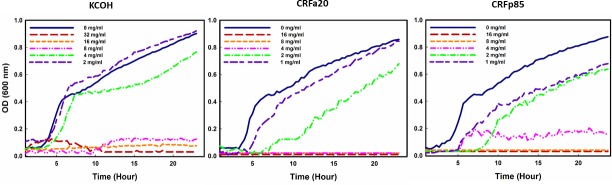
Concentration-dependent growth inhibition of *Salmonella* Enteritidis ABBSB1004-1 in CAMHB containing ethanolic extract from cranberry pomaces (KCOH) and two of it sub-fractions anthocyanins (CRFa20) and non-anthocyanin polyphenols/flavonols (CRFp85).

All tested *Salmonella* serovars Enteritidis, Typhimurium and Heidelberg isolates showed a similar susceptibility profile to tested products regardless their antimicrobial resistance pattern. The MIC and MBC values of KCOH were 8 and 16 mg/ml, respectively against all tested isolates. Reduced values of MIC (4 mg/ml) and MBC (4 mg/ml) were obtained with CRFp85 sub-fractions; these values were 4 and 8 mg/ml, respectively for CRFa20 (Table 2 and Fig 2). CRFa20 and CRFp85 induced up to seven hours of lag phase of growth and a strong growth rate inhibition of *Salmonella* Typhimurium, Enteritidis and Heidelberg.

**Table 2:** Minimal inhibitory concentrations (MICs: mg/ml) and minimal bactericidal concentrations (MBCs: mg/ml) of cranberry pomace extracts (KCOH) and its sub-fractions CRFa20 (anthocyanins) and CRFa85 (polyphenols) against different serovars of *Salmonella*.

Serovars	Isolates	KCOH		CRFa20		CRFp85	
		MIC	MBC	MIC	MBC	MIC	MBC
**Enteritidis**	ABBSB1004-1	8	16	4	8	4	4
**Typhimurium**	ABBSB1218-1	8	16	4	8	4	4
**Heidelberg**	SALB-159-4	8	16	4	8	4	4

### Differentially expressed genes (DEGs) in response to KCOH

**Fig 3:**
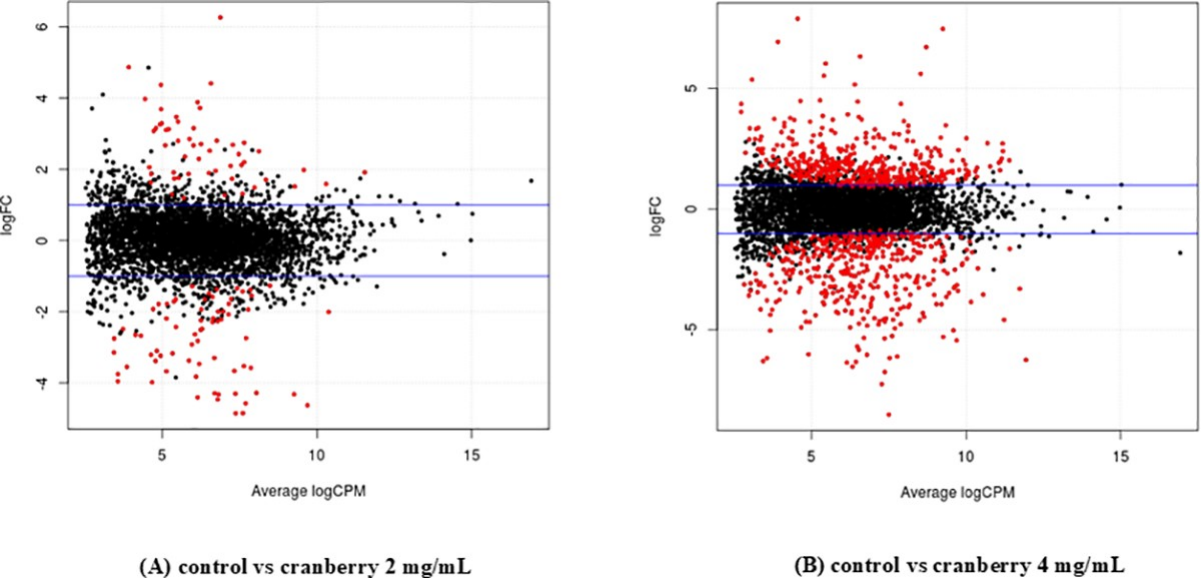
Scatterplot of the differential gene expression levels of *S*. Enteritidis ABBSB1004-1. The horizontal (x-axis) coordinates represent the log_2_-transformed CPM values for each gene, and the vertical (y-axis) coordinates represent the log_2_-transformed fold changes for each gene in (A) control vs cranberry 2 mg/mL; (B) control vs cranberry 4 mg/mL exposure. Red dots represent DEGs.

In this study, *S*. Enteritidis ABBSB1004-1 was used for strand-specific RNA sequencing after 4 hours exposure to 2 mg/ml (1/4 MIC) and 4 mg/ml (1/2 MIC) of KCOH. No growth inhibition was observed with these two sub-MICs of KCHO in four hours. Differentially expressed genes (DEGs) were estimated by comparing transcripts from culture in these two concentrations to that of a control culture. Overall, these cultures on *S*. Enteritidis in CAMHB resulted in the expression of 4182 genes. Comparison of significant DEGs between culture conditions were based on a false discovery rate (FDR) < 0.05 and fold-change ≥ 2 (Fig 3).

**Fig 4:**
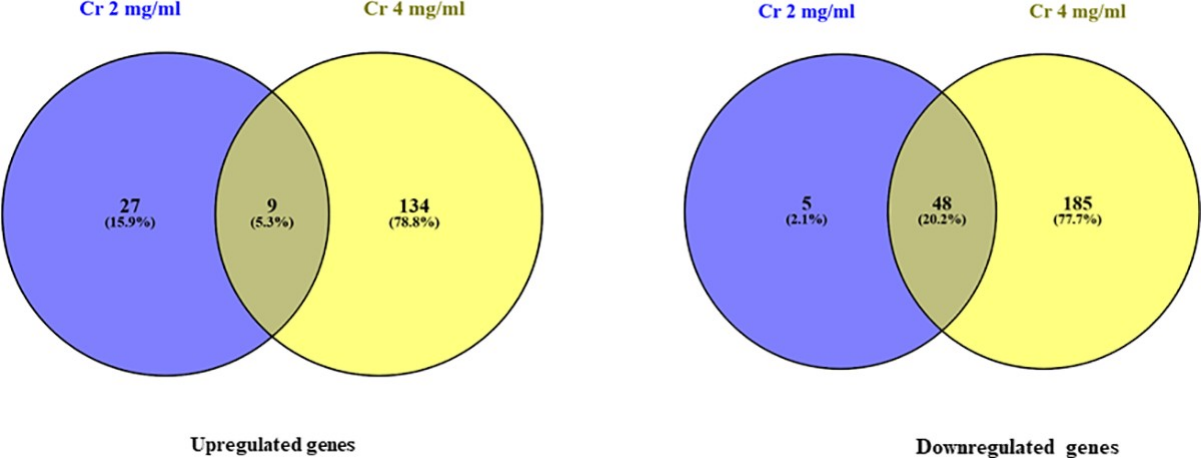
Venn diagram showing overlap of differentially expressed genes following exposure to 2 and 4 mg/mL KCOH.

**Fig 5:**
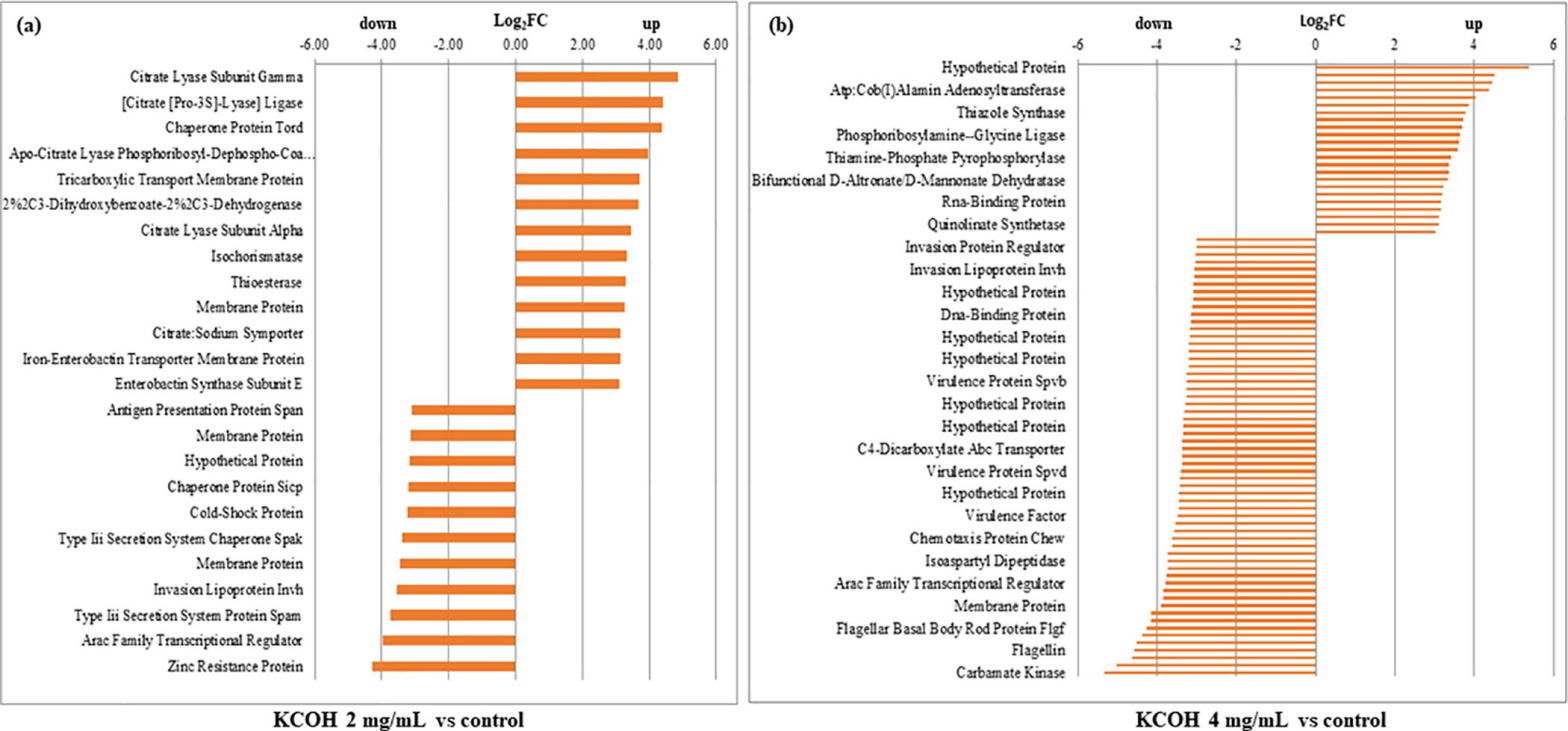
**(a)** Differentially Expressed Genes (DEGs) (≥3 Log-Fold upregulated or downregulated genes) in *S*. Enteritidis ABBSB1004-1 at KCOH 2 mg/mL vs control; **(b)** Differentially Expressed Genes (DEGs) (≥3 Log-Fold upregulated or downregulated genes) in *S*. Enteritidis ABBSB1004-1 at KCOH 4mg/mL vs control.

After exposure to 2 mg/ml KCOH, 89 (2%) of 4182 genes were differentially expressed, among which 53 and 36 genes were downregulated and upregulated, respectively (Fig 4). Culture of *S*. Enteritidis in 4 mg/ml KCOH induced a greater number of modulated (up and down regulation) genes compared to control. KCOH at 4 mg/ml resulted in the modulation of 376 (9%) of total 4182 genes, among which 233 genes were downregulated while 143 were upregulated (Fig 4). Overall 57 (>2%) genes were significantly differentially expressed in both concentrations, where 48 and 9 genes were down and upregulated; respectively (Fig 4). Some of the major differentially expressed genes (more than >3-fold) are presented in the (Fig 5A and 5B).

**Fig 6:**
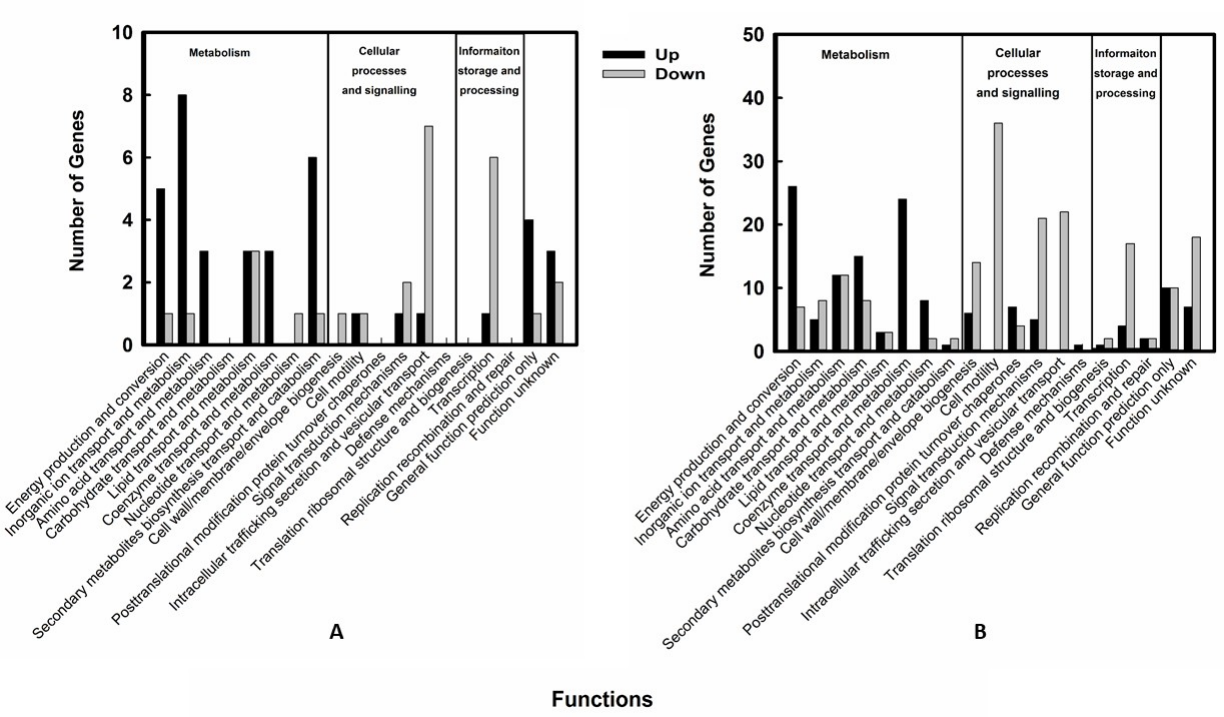
Overview of the differentially expressed genes according to their function. Genes significantly upregulated or downregulated at different concentrations of KCOH (A) control vs KCOH at 2 mg/mL and (B) control vs KCOH at 4 mg/mL in *S*. Enteritidis were grouped according to their Clusters of orthologous groups (COGs) functional categories. Since one gene can be classified into more than one COG class, the total number of COG assignments is greater than the number of differentially expressed genes.

To further clarify the functions of modulated genes, they were categorised based on their orthologous (COG) relationships into three major categories: 1) metabolism; 2) cellular processing and signalling; and 3) information storage and processing. The up and down regulated genes were further classified into 18 different sub-categories associated with similar functions (Fig 6). The modulated genes are described below.

#### Intracellular trafficking secretion and vesicular transport: *Salmonella* pathogenicity islands (SPI)

**Fig 7:**
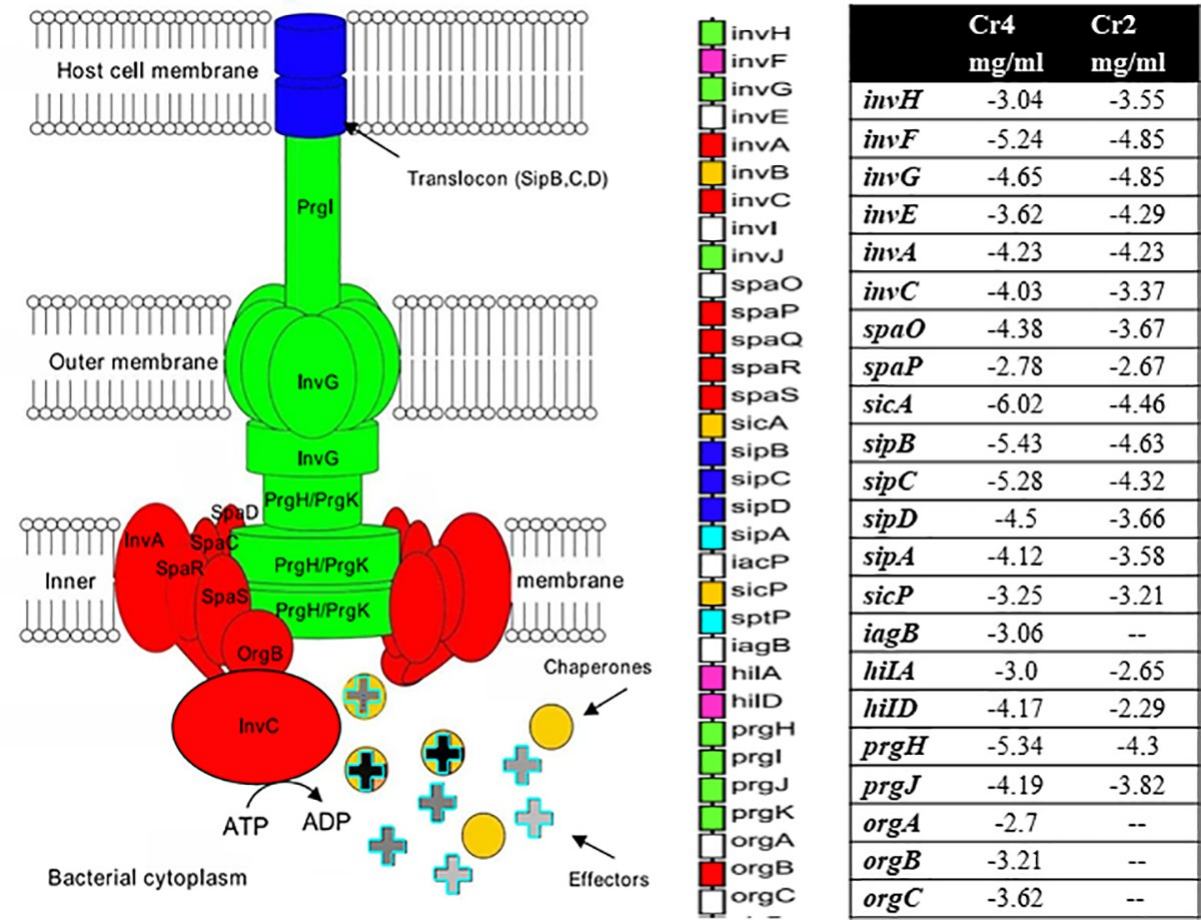
Schematic representation of SPI-1 island encoding the T3SS-1 proteins showing DEGs (in the chart) in response to KCOH. T3SS-1 is a supramolecular complexes that plays a major role in the virulence of *Salmonella* by injecting bacterial protein effectors directly into host cells (adopted from [37]).

Exposure of *S*. Enteritidis to both concentrations (2 and 4 mg/ml) of KCOH led to the downregulation of genes encoding the inner (*prgH* and *prgK*) and the outer *invG* ring proteins along with *prgI* for the needle protein. Expression of the *sipBCD* genes encoding proteins associated with the translocation of polypeptides across membranes (translocon) and the *sipA* gene encoding their effector proteins were also down regulated by both concentrations of KCOH (Fig 7). During translocation across the intestinal epithelium, *Salmonella* employs the *SsaKLMNOPQRSTUV* operon [*Ssa* (secretion system apparatus)] that encodes structural components of SPI-2. Interestingly, all 13 *ssa* genes belong to SPI-2 locus were upregulated after treatment with 4 mg/ml KCOH.

#### Cellular processes and signalling: Cell motility

Exposure to the evaluated KCOH led to downregulation of several genes essential for *Salmonella* virulence, including those associated with motility, chemotaxis, and adherence. After exposure to 4 mg/ml, the flagellar structural genes *fliA*, *fliL*,*and fliM*, as well as *flgC flgD*, *flgE*, *flgF*, *flgG*, *flgH flgK*, *and flgL* (log_2_ fold-change between 4.5 to 2.0) required for cell motility in the cellular processes and signalling category were downregulated. In addition, two essential genes for flagellar rotation (*motAB)* and several chemotactic genes such as chemotaxis protein *CheA*, *CheR*, *CheW*, *CheV*, *CheY*, *CheZ* were downregulated with 4 mg/ml KCOH.

Both 2 and 4 mg/ml of KCOH treatments led to downregulation of transcriptional regulators (*hilA*, *hilD)* and invasion (*invH*, *invF*, *invG*) genes. Exposure to 4 mg/ml of KCOH downregulated several genes from the *spaKNRPTMO* operon, with the most downregulation proteins being SpaO (log_2_ fold-change = 4.4) and OrgA (log_2_ fold-change = 2.7).

#### Metal transport proteins

To regulate its metal homeostasis, *Salmonella* employs several transcriptional regulators for its adaptation to changing environments. Many of these regulators belong to genes categorised in “Inorganic ion transport and metabolism” or “Secondary metabolites biosynthesis transport and catabolism groups” COG functional categories. Our results show that iron-regulated genes, including those associated with enterobactin (catecholate siderophore) synthesis (*entE*, *entF*) and transport (ABC transporters and permerases), as well as ferrous uptake (*feoB*), were upregulated by KCOH. The upregulation of these iron-related genes indicates that KCOH induces iron starvation. Moreover, in both concentrations (2 and 4 mg/ml) of KCOH, *zraP*, which is responsible for zinc resistance, was downregulated. In addition, exposure to the higher concentration (4 mg/ml) of KCOH downregulated genes encoding the copper-binding protein required for copper tolerance, which is also involved in resistance to heavy metals.

#### Metabolism

RNA-seq revealed several genes related to “energy production and conversion”, “carbohydrate transport and metabolism” and “coenzyme transport and metabolism” were upregulated in response to KCOH. For example, citrate lyase, nitrate reductase, aldehyde reductase, hydroxylamine reductase, succinate dehydrogenase, lactate dehydrogenase, NADH dehydrogenase were all upregulated at KCOH 4 mg/ml but not at 2 mg/ml.

Numerous genes associated with amino acid biosynthetic pathways, including the carbamoyl phosphate synthase *carB* (2.39 log fold) gene were upregulated. Moreover, 6.9 to 4.4 log_2_-fold change upregulation of succinate dehydrogenase and nitrate reductase including citrate lyase subunit alpha (the alpha subunit catalyzes the formation of (3S)-citryl-CoA from acetyl-CoA and citrate), citrate lyase subunit beta (catalyzes the formation of acetate and oxaloacetate from citrate), and citrate lyase subunit gamma were also observed.

In contrast, genes related to the tetrathionate reduction and ethanolamine utilization (*eutP*, *eutQ*) were downregulated in response to KCOH suggesting that the cranberry pomace extract could interrupt pathways critical for *Salmonella* survival. A set of genes related to the “catalyse of the formation of amide derivatives” were upregulated (~3.5 folds). Upregulation of *purH* indicates the important role of purine during metabolism; however other genes (*purT*, *purl*,*purM*) were not expressed during exposure to 4 mg/ml KCOH.

#### Defense mechanism

Along with the categories described above, a few critical genes involved in defense mechanism were induced by the treatment of *S*. Enteritidis with 4 mg/ml of KCOH. Among them, the gene coding for the heat shock protein belonging to the “Post-translational modification protein turnover chaperones” category was upregulated (2.02 log_2_-fold). Interestingly, genes related to the cold shock proteins in the “Transcription” category were downregulated by both concentrations (2 mg/ml: -3.18 log_2_-fold; 4 mg/ml: -4.67 log_2_-fold) of KCOH. Except *emrB* and *mdtH*, no modulations of efflux pumps or antibiotic resistance genes were observed. These multidrug resistance genes (*emrB* and *mdtH*: 2.52 and 2.98-log fold, respectively) were upregulated at 4 mg/ml of KCOH.

### Confirmation with RT-qPCR

**Fig 8:**
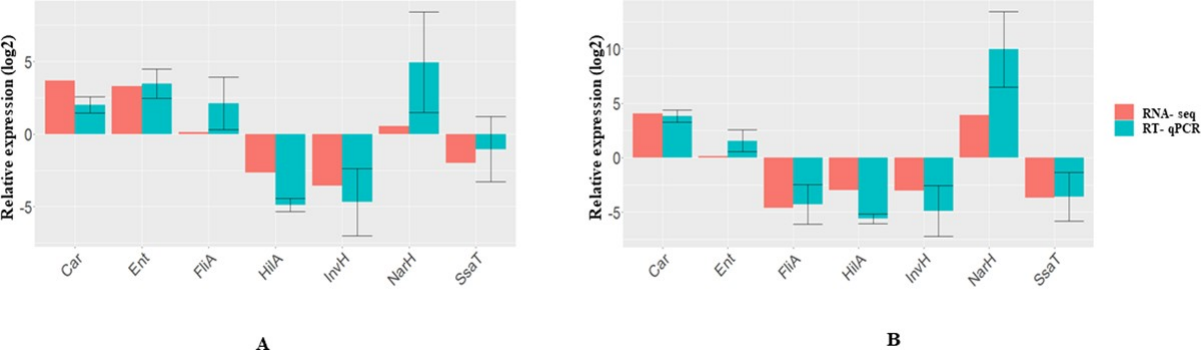
Validation of the RNAseq data for selected genes by RT-qPCR. RT-qPCR data confirmed the expression trends observed in the RNA-seq data for seven genes in KCOH 2 and 4 mg/mL compared to control, where *rpoD* used as a reference gene.

To validate the RNA-seq results, seven genes (Table 1) showing significantly different transcription levels in response to KCOH were analyzed with RT-qPCR. RNA-seq and RT-qPCR results after exposure to 2 mg/ml KCOH, were in agreement for all tested genes except for *fliA* (flagellar biosynthesis sigma factor) *and narH* (nitrate reductase). Accordingly, in culture with 2 mg/ml KCOH, a slight *fliA* and *narH* modulation was observed by RNA-seq however, these genes were highly upregulated using RT-qPCR. Regarding exposure to 4 mg/ml KCOH, *hilA*, *invH*, *ent*, *flliA*, *ssaT* were down-regulated; and *car* and *narH* genes were upregulated both in RNA-seq and RT-qPCR, confirming the reliability and accuracy of the RNA-seq expression analysis (Fig 8).

## Discussion

Despite substantial efforts deployed by industry, the control of *Salmonella* in poultry to improve food safety remains challenging. The control of *S*. *enterica* serovars is difficult due to the ability of this pathogen to survive, spread, and persist in all levels of the food chain. The clear picture of *Salmonella* infection in different host is yet to be confirmed however it is principally composed of 3 steps; (1) attachment and adhesion to host cell surface, (2) production of factors allowing host cell invasion and overcoming host defense mechanism and (3) multiplication inside the host cell [32]. In this study, we have examined the antibacterial activities of KCOH and two of its sub-fractions (CRFa20 and CRFp85) against different serovars of *Salmonella* and explored its effects on the transcriptional profile of *S*. Enteritidis. We previously discussed the use of berry fruit products as novel approaches to control pathogenic bacteria including *Salmonella* in the food industry [15]. The antibacterial activity of cranberry extracts against both gram-positive and gram-negative bacteria has also been investigated by several researchers [22, 33]. We reported that KCOH is a rich source of organic acids, such as citric, malic and quinic acids and contains 94.75% carbohydrate on a dry matter basis [19]. In the present study, each studied cranberry extract showed similar MICs against the three tested *Salmonella* serovars with sub-fractions CRFa20 and CRFp85 being the most active (reduced MIC values) compared to KCOH from which they derived. It is not clear, whether the observed antibacterial activity of KCOH is from CRFa20 (anthocyanins) or CRFp85 (flavonols); however, CRFp85 seems to be more active with its lower MBC value (Table 2). Quercetin and myricetin, the two most abundant components in our flavonoid fraction, are well known for their bivalent metal cations chelation. Due to the presence of *ortho* dihydroxyl phenolic and hydroxyketo groups, these flavonols can chelate “free” copper and iron ions from the environment, which could be the reason for the increased growth inhibition by CRFp85 [34].

*Salmonella* possess lipopolysaccharides and a number of other outer membrane components such as fimbria in order to colonize and cause systemic infection in their hosts [12]. There are more than 40 genes involved in flagellar biosynthesis controlled by a regulatory cascade, which is initiated by the production of *FlhDC* [35]. In the present study, several genes associated with the motility, chemotaxis, and invasion of *Salmonella* Enteritidis (Table 3) were modulated after exposure to sub-MICs of KCOH. The flagellar basal body operon *flgBCDEFG* and flagella biosynthesis factors such as *fliA* and *fijB* were downregulated. This trend was also observed by other groups [33, 36] who demonstrated the downregulation of flagellum-mediated motility (*fliC*, *fla*) of uropathogenic *E*. *coli* and *P*. *mirabilis* in presence of cranberry proanthocyanidins. Following COG analysis, we found all 33 genes involved in cell motility were down-regulated by 4 mg/ml of KCOH, which might be an energy conservation strategy for *S*. *enterica* under stress.

**Table 3:** List of genes regulated after the exposure to sub-inhibitory concentrations (2 and 4 mg/ml) of KCOH in *S*. Enteritidis.

Gene	Function/product	LogFC
**Flagella**		
*fliA*	flagellar biosynthesis sigma factor	-4.63833
*flgL*	flagellar hook protein FlgL	-2.99028
*flgE*	flagellar hook protein FlgE	-4.51268
	flagellar biosynthesis protein FliO	-3.75083
*flgF*	flagellar basal body rod protein FlgF	-4.25327
*fliM*	flagellar motor switch protein FliM	-2.54921
*fliL*	flagellar basal body-associated protein FliL	-2.63933
	flagellar protein FliD	-1.66659
*flgG*	flagellar basal body rod protein FlgG	-3.21002
*flgC*	flagellar basal body rod protein FlgC	-3.42003
*flgH*	flagellar basal body L-ring protein	-2.78366
	flagellar biosynthesis protein FliT	-1.79537
*fliS*	flagellar protein FliS	-1.94095
*fliP*	flagellar biosynthesis protein flip	-1.62597
*flgD*	flagellar basal body rod modification protein	-3.2572
	flagellar brake protein	-1.48432
	flagella biosynthesis protein FliZ	-1.40332
*fliN*	flagellar motor switch protein FliN	-2.08972
*flgI*	flagellar basal body P-ring biosynthesis protein FlgA	-1.87598
	flagellar biosynthesis protein FlgN	-2.44643
	flagellar protein flhE	0.503612
	cell density-dependent motility repressor	-3.17148
**Chemotaxis**		
*CheW*	chemotaxis protein	-3.60626
	chemotaxis protein	-3.84947
*CheV*	chemotaxis protein	-2.02205
	chemotaxis protein	-2.3658
*CheY*	chemotaxis protein	-2.67638
*CheZ*	chemotaxis protein	-2.00699
	chemotaxis protein	-1.42596
*CheY*	chemotaxis protein	-0.17472
	chemotaxis protein	0.088919
	chemotaxis protein	-3.46467
*CheW*	chemotaxis protein	-3.60626
	chemotaxis protein	-3.84947
*CheV*	chemotaxis protein	-2.02205
	chemotaxis protein	-3.46467
**Outer membrane protein**		
*LolB*	outer membrane lipoprotein LolB	-1.29
	type III secretion system outer membrane pore InvG	-4.85
**Iron transportation**		
*entF*	enterobactin synthase subunit F	2.42
*entE*	enterobactin synthase subunit E	3.1
	iron-enterobactin transporter ATP-binding protein	1.74
	iron-enterobactin transporter membrane protein	3.12
*entE*	enterobactin synthase subunit E	3.1
	iron ABC transporter	1.91
	iron ABC transporter permease	3.07
	ferrous iron transporter C	1.76
*feoB*	ferrous iron transporter B	1.51
	iron-enterobactin ABC transporter substrate-binding protein	2.35
	isochorismatase	3.33
	2%2C3-dihydroxybenzoate-2%2C3-dehydrogenase	3.68
	thioesterase	3.29
**Carbohydrate metabolism**		
eno	(enolase) catalyzes the formation of phosphoenolpyruvate from 2-phospho-D-glycerate in glycolysis	-0.90
gapA	(glyceraldehyde-3-phosphate dehydrogenase) required for glycolysis; catalyzes the formation of 3-phospho-D-glyceroyl phosphate from D-glyceraldehyde 3-phosphate	-0.50
	citrate lyase subunit alpha	3.47
	citrate lyase subunit gamma	4.86
	[citrate [pro-3S]-lyase] ligase	4.41
	citrate:sodium symporter	3.15
	(6-phosphofructokinase) catalyzes the formation of D-fructose 1,6-bisphosphate from D-fructose 6-phosphate in glycolysis	-0.45679

The expression of invasion genes is tightly regulated by environmental conditions such as oxygen, pH and osmolarity. *Salmonella* invades host cells by using T3SS encoded by SPI-1. Type-III secretion systems fulfil two significantly different roles in bacteria; first they act as an assembly and export system in the production of the flagellum (Flagellar- or F-T3SSs) and second, they translocate effector proteins into host cells [37]. Translocation of effector proteins requires an exportation apparatus controlled by *spaP*, *spaQ*, *spaR*, *spaS*, *invA*, *invC* and *orgB* [38]. More than 25 genes are required for the host-invasion by *S*. Typhimurium. Our RNA-seq results showed that 15 genes present in SPI-1 were down-regulated in the presence of sub-inhibitory concentrations of KCOH. KCOH at 2 mg/ml significantly down-regulated the expression of *spa* genes (*spaK*, *spaM* and *spaN*) belonging to the *inv-spa* complex. Downregulation of both *spaM* and *spaK* involved in the functionality of T3SS [39] suggests that KCOH may restrict the entry of *Salmonella* into the host cell. Interestingly, similar regulatory action was not observed by Harmidy et al. [40], who reported that cranberry proanthocyanidin had no detectable effect on the T3SS. The discrepancy between our study and those found in the literature could be explained by the nature and the doses of cranberry fractions used.

The *hilA* gene encodes an OmpR/ToxR transcriptional regulator that activates expression of *Salmonella* invasion genes in response to both environmental and genetic regulatory factors [41]. Boddicker et al. [42] reported that the absence of *hilD*, an AraC/XylS regulator, resulted in low-level expression of *hilA*, suggesting that *hilD* is required for activation of *hilA* expression. In the present study, exposure of *S*. Enteritidis to 2 or 4 mg/ml of KCOH resulted in the downregulation of both *hilD* and *hilA*. Moreover, downregulation of a set of invasion genes *invAEFGH* and upregulation of *prgHIJK* were observed after exposure of Enteritidis to KCOH at 4 mg/ml. Similar gene expression patterns were observed when *Salmonella* was exposed to bile or cationic peptides in the proximal small intestine or macrophage of host cell, respectively [38]. Reduced expression of genes involved in transcription regulators (*hilA*, *hilC*) were also reported with blueberry and blackberry pomace extracts against *S*. Typhimurium [43].

Six T3SS-1 effectors, including *sipA*, *sopA*, *sopB*, *sopD*, *sopE*, and *sopE2*, are required for the epithelial invasion of *Salmonella* [44]. Introduction of either *sip* or any of the mentioned *sop* genes increased the invasiveness of the *sipA*, *sopABDE2*. In the present study, exposure of *S*. Enteritidis to 4 mg/ml KCOH significantly downregulated *sopD2*, indicating that this cranberry pomace extract could affect the invasion of host cells by Enteritidis. Accordingly, the condensed tannins of cranberry extracts most commonly known as proanthocyanidins (cPACs) has been reported to reduce the hydrophobicity of bacterial cell surfaces and interfere with their attachment to host cellular or biomaterial surfaces [45]. A similar anti-motility trend was also observed with cranberry pomace extracts against *Enterococcus faecalis*, *Escherichia coli*, *Pseudomonas aeruginosa* and *Proteus mirabilis* [45–47].

Iron is an essential metal in nearly all living organisms, serving as a cofactor for proteins involved in redox chemistry and electron transport. Besides, iron availability is an important determinant of virulence. Iron-deficient mice showed resistance to *S*. Typhimurium infection, and an increased systemic iron concentrations correlated with bacterial growth, adhesion, invasion, and lethality [48]. Several publications reported that antibacterial activities of cranberry proanthocyanidins (cPACs) were linked to metal chelation. Cranberry constituents such as flavonoids and phenolic compounds could possibly act as metals including iron chelators [20]. Under iron restriction, bacteria typically synthesize and secrete high-affinity ferric chelators called siderophores, which solubilize exogenous iron, making it available for uptake [49]. *Salmonella* produces two types of catecholate siderophore, enterobactin and salmochelin. In the present study, the up-regulation of *entAEH* (enterobactin biosynthesis and transportation) after exposure of *S*. Enteritidis to 2 mg/ml KCOH supported that PACs induce an iron limitation state in bacterium [33]. The hydroxyl groups of proanthocyanidins interact with iron ions using “iron binding motifs” present in the flavanol structure [50].

Citrate can be utilized by *Salmonella* as a carbon and energy source using several catabolic fermentative pathways involving genes located in two divergently transcribed operons, *citCDEFG* and *citS-oadGAB-citAB*, whose expression is modulated by the citrate-sensing CitA/CitB two-component system [51]. CitAB has been shown to contribute to *Vibrio cholerae* competitiveness within the gut microbiota; however, the role of citrate fermentation in pathogenic bacteria during infection is not clear [52]. Since, cranberry extract is a source of citrate, it may serve as a carbon source during the TCA cycle, to play an important regulatory molecule in the control of glycolysis and lipid metabolism [37]. As an iron-chelator, citrate is involved in the homeostasis of iron in the pathogen. Interestingly, both 2 and 4 mg/ml of KCOH upregulated the *citBCDFNX* operon. Under stressful conditions, the export pump *IctE* (iron citrate efflux transporter, former called *MdtD*) transports iron chelated with citrate out of the cell, decreases harmful cellular iron content and reduces the growth of *Salmonella*. Bacteria usually employ a variety of metal uptake and export systems and finely regulate metal homeostasis by numerous transcriptional regulators, allowing them to adapt to changing environmental conditions. We observed the transcriptional changes of several iron, zinc, manganese and copper uptake systems genes, supporting the notion that KCOH significantly affects the virulence of pathogenic bacteria [53].

Nutrient limitation by the host and nutrient acquisition by pathogenic bacteria are crucial processes in the pathogenesis of bacterial infectious diseases. Bacteria have developed sophisticated acquisition systems to scavenge essential metals from the environment, which are up-regulated during metal starvation. Zinc plays a role in bacterial gene expression, general cellular metabolism and acts as a cofactor of virulence factors. Procuring enough zinc to sustain growth during infection is a considerable challenge for bacterial pathogens. While zinc is an essential nutrient, excess zinc is toxic to the cell, possibly through inhibition of key enzymes and competition with other relevant metal ions [54]. Our transcriptomic results showed a downregulation of *zraP*, indicating that S. Enteritidis was ensuring enough concentrations of zinc to fulfill essential functions while limiting concentration to prevent toxic effects.

*Salmonella* is a facultative anaerobe that transitions between aerobic and anaerobic growth by modulating its central metabolic pathway. In anaerobic condition, *Salmonella* performs fermentation by overexpressing nitrate as an electron acceptor. At 4 mg/ml of KCOH, we observed a significant upregulation of several nitrate reductase genes (*narHLZ*) probably because of the anaerobiosis or nitrate reduction; however, we did not see any expression of formate lyase, another principle enzyme for anaerobic fermentation [55]. When grown in a glucose-rich environment, *Salmonella* utilizes glycolysis for cellular energy production instead of using the full TCA cycle. KCOH exposure might create an environment suitable for TCA cycle; leading to the upregulation of a set of TCA cycle enzymes to meet the cellular demand for pyruvate and acetyl-CoA instead of glycolysis enzymes [56]. Downregulation of *aceK*, encoding for iso-citrate dehydrogenase, usually occurs when cells grown on glucose, then phosphorylated in presence of acetate or ethanol. These acetates could be the by-products of glycolysis. During colonization, *Salmonella* utilizes a wide range of carbohydrate sources e.g. glucose, lactose, pyruvate, L-lactate, melibiose, ascorbate as well as other scarce carbon compounds such as sialic acid, ethanolamine from the host mucosa [57]. We observed that KCOH at 4 mg/ml downregulated several carbohydrate transport and metabolism proteins such as mannose transporter, maltose transporters, xylanase deacetylase, formamide-L-arabinose-phospho-UDP deformylase. Moreover, the downregulation of *eutPQ* gene signifies that KCOH reduce the bacterial activity to utilize ethanolamine as an energy source. In host intestine, *Salmonella* can utilize ethanolamine as a sole source of carbon, nitrogen and energy by utilizing tetrathionate as a terminal electron acceptor, which is not available to most members of the host microbiota [57]. The highest upregulation for the energy metabolism was found for the citrate lyase subunit alpha, beta and gamma after the nitrate reductase expressions. Moreover upregulation of *sdhA* and *sucC* specifies the relationship of TCA cycle and virulence [37]. The upregulation of these enzymes indicates that in the presence of KCOH, *Salmonella* most probably utilize citrate as carbon and energy sources for the acetate formation by utilizing anaerobic citrate fermentation metabolic pathway. As a consequence, an upregulation of NADH:ubiquinone oxidoreductase enzyme was observed in the present study. During anaerobic fermentation, bacteria requires reducing agents to convert citrate into cellular materials [58]. During KCOH exposure, quinone may serve as an electron carrier between a membrane bound formate dehydrogenase and NADH, resulting in the upregulation of FMN-dependent NADH-azoreductase, NADH dehydrogenase and NADH:ubiquinone oxidoreductase enzymes.

The present study explored the antimicrobial activities of cranberry pomace extracts against *Salmonella enterica* from chicken and revealed antibacterial actions attributed to several genes involved in motility, in uptake of metals including iron, and in invasion (SPI-1 and SP2-1) of host cells. These results provide for the first time the framework for future studies to develop cranberry extracts as natural compounds to fight against the most important *S*. *enterica* serovars found in poultry.
